# The Role of Epicardial and Visceral Fat in Severe Respiratory Infections: Associations With Adverse Outcomes

**DOI:** 10.1111/cob.70089

**Published:** 2026-06-10

**Authors:** Rebecca Baumgartner, Jéssica A. de Paula, Cleverson A. Leitão, Gabriela Lazzaron‐Slob, Estela I. Rabito, Emilton L. Junior

**Affiliations:** ^1^ Postgraduate Program in Internal Medicine and Health Sciences at Federal University of Paraná Curitiba Paraná Brazil; ^2^ Clinical Nutrition Unit Hospital de Clínicas, Federal University of Paraná Curitiba Paraná Brazil; ^3^ Postgraduate Program in Food and Nutrition at the Federal University of Paraná Curitiba Paraná Brazil; ^4^ Department of Nutrition, Federal University of Paraná Curitiba Paraná Brazil

**Keywords:** COVID‐19, epicardial adipose tissue, mortality, visceral adipose tissue

## Abstract

Excess adipose tissue has been extensively studied due to its role in the development of chronic diseases and inflammatory conditions. Obesity and visceral adipose tissue (VAT, fat stored within the abdominal cavity and around organs) are associated with increased susceptibility to respiratory infections and chronic low‐grade systemic inflammation. This study investigates the relationship among VAT, epicardial adipose tissue (EAT, fat surrounding the heart), and mortality in critically ill patients diagnosed with COVID‐19, aiming to identify which measure is more strongly associated with adverse outcomes. A retrospective observational cohort study was conducted with patients admitted to the Intensive Care Unit (ICU) who underwent chest computed tomography (CT). Measurements of VAT and EAT were obtained from contrast‐free CT images. Statistical analysis included the Mann–Whitney *U* test and logistic regression. Individuals who died had a larger EAT area. In patients with BMI < 30 Kg/m^2^, an increase in EAT was associated with a higher risk of mortality (OR = 1.06, 95% CI 1.015–1.107, *p* < 0.01). EAT area measurement was associated with mortality risk in critically ill individuals without obesity (BMI < 30 kg/m^2^) diagnosed with COVID‐19 and may serve as a prognostic marker for risk stratification in this population.

## Key Points

- What is already known about this subject
  ○ Excess adipose tissue (body fat) contributes to a state of chronic low‐grade inflammation, a persistent but mild condition.
  ○ Epicardial adipose tissue (fat surrounding the heart) has been linked, through mechanisms not yet fully understood, to the progression of infectious respiratory diseases.
  ○ Individuals with obesity (excess body fat) may exhibit a survival advantage when admitted to intensive care units (ICUs, specialized hospital wards for critically ill patients).
- What this study adds
  ○ Measurement of epicardial adipose tissue may be more informative than visceral adipose tissue (visceral fat) measurement as a prognostic marker associated with mortality in severe respiratory infections; however, these findings should be interpreted as associations pending validation in prospective studies with comprehensive confounder adjustment.
  ○ Increased epicardial adipose tissue in individuals without obesity is more strongly associated with mortality.
  ○ Using computed tomography (CT) images to assess different types of visceral fat may serve as a complementary prognostic tool for risk stratification, subject to confirmation in studies with more rigorous confounder control.

## 1 Introduction

Excess adipose tissue has been studied for its role in chronic diseases and inflammation [1]. Since body composition refers to the proportions of fat, muscle, and other tissues, it affects nutritional status, inflammatory response, and metabolic activity [2, 3].

Individuals with obesity are more prone to respiratory infections and persistent, mild inflammation [2, 4]. As a result, compared to people of normal weight, they have a higher risk of admission to the Intensive Care Unit (ICU), lower vaccine effectiveness, more severe disease, and a higher chance of death. These outcomes were evident during the COVID‐19 [5, 6] and 2009 Influenza A/H1N1 pandemics [7].

Body mass index (BMI) is widely used for diagnosing obesity. BMI is a numerical value calculated from an individual's weight and height, but it does not accurately show body fat mass at the individual level [8]. Importantly, a BMI below 30 kg/m^2^ does not exclude excess visceral or ectopic fat accumulation. Some individuals with a BMI below the clinical obesity threshold may exhibit a metabolically unhealthy body composition—sometimes referred to as metabolically unhealthy normal weight—characterized by disproportionate visceral adiposity and pro‐inflammatory activity despite not meeting conventional obesity criteria.

Different locations of visceral fat accumulation can affect physiological responses differently. Abdominal visceral adipose tissue (VAT) is linked to cardiovascular disease (CVD), diabetes mellitus (DM), hypertension (HTN), and systemic inflammation [2, 9, 10, 11, 12]. Similarly, epicardial adipose tissue (EAT), the visceral fat surrounding the heart, is a measurable risk factor for mortality and disease severity. EAT has unique metabolic and functional features relevant to infectious disease pathogenesis [13, 14].

These associations highlight the importance of accurately assessing visceral fat, which is stored within the abdominal cavity around internal organs. This helps to better understand its role in disease outcomes. However, evaluating visceral fat requires imaging, such as computed tomography (CT), an advanced x‐ray technique that produces detailed cross‐sectional images of the body. During COVID‐19, CT scans were widely used in critically ill COVID‐19 patients to assess lung damage, providing an opportunity to measure visceral adipose tissue [15].

Studying the effect of specific visceral fat areas on critically ill COVID‐19 patients is crucial. It helps to identify those at higher risk of mortality early. Also, understanding visceral fat's role in disease severity may motivate lifestyle changes and the pursuit of a healthier body composition. This study aimed to verify the relationship among EAT, VAT and mortality risk in this group. We sought to identify which measure is most strongly associated with adverse clinical outcomes.

## 2 Material and Methods

### 2.1 Study Design and Population

This is a retrospective cohort observational study approved by the Human Research Ethics Committee (no. 35738620.8.0000.0096). Patients aged 18 years or older, of both sexes, were included if they were admitted to the ICU of a Public University Hospital in Southern Brazil. They needed a positive SARS‐CoV‐2 reverse transcription polymerase chain reaction (RT‐PCR) test and a chest computed tomography (CT) scan performed within 72 h of admission.

The sample size was selected from eligible ICU patients between March 2020 and December 2021. All patients who met the inclusion criteria in this period were included and followed until death or discharge to the ward. Exclusion criteria were incomplete medical records, contrast‐enhanced CT scans compromising image quality, and CT missing the required evaluation area.

### 2.2 Data Collection

We collected data from electronic and physical patient records. We also used nutritional assessment and monitoring forms completed by nutritionists. Our group created a standardized data collection form with predefined variables and operational definitions to ensure consistency and reduce information bias. Variables recorded included days on mechanical ventilation (MV), ICU length of stay, anthropometric measurements, mortality, and comorbidities such as DM, dyslipidemia (DP), CVD, and HTN.

### 2.3 Anthropometric Measurements

Anthropometric measurements were collected within the first 72 h of admission. These measurements included weight, height, mid‐upper arm circumference (the distance around the upper arm at the midpoint between the shoulder and elbow), and knee height (the distance from the heel to the top of the knee when the leg is bent at a right angle). All measurements were taken using a non‐elastic tape, accurate to 0.1 cm. If direct values were not possible, weight and height were estimated using validated predictive equations, as recommended for COVID‐19 nutritional assessment [16, 17, 18]. Patients were classified as having obesity (BMI ≥ 30 kg/m^2^; BMI is Body Mass Index, calculated as weight in kilograms divided by height in meters squared) or as not having obesity (BMI < 30 kg/m^2^). This BMI‐based classification, while widely used and clinically practical, has recognized limitations: a BMI below 30 kg/m^2^ does not preclude the presence of excess visceral or ectopic fat, and individual heterogeneity in body fat distribution is not captured by this metric alone.

### 2.4 Visceral Fat Measurements

Non‐contrast chest CT images were used to quantify VAT and EAT. Images were acquired with a 64‐slice scanner (Toshiba Aquilion 64 TSX 101A, Tokyo, Japan). Scanning parameters: tube voltage 120 kV, tube current 150–550 mA, rotation time 0.4 s, pitch 1.375 mm/rotation, and 64‐channel detector collimated at 0.625 mm. Images were reconstructed with slice thicknesses of 0.625 mm for the lungs and 3 mm for the mediastinum.

GE Healthcare Ready View software was used for image processing. A single radiologist with 7 years of experience performed all measurements. EAT was measured at the axial slice level of the left coronary artery trunk using existing, validated methods [19, 20]. VAT was measured in the midplane of the T12/L1 intervertebral disc space [21].

**FIGURE 1:**
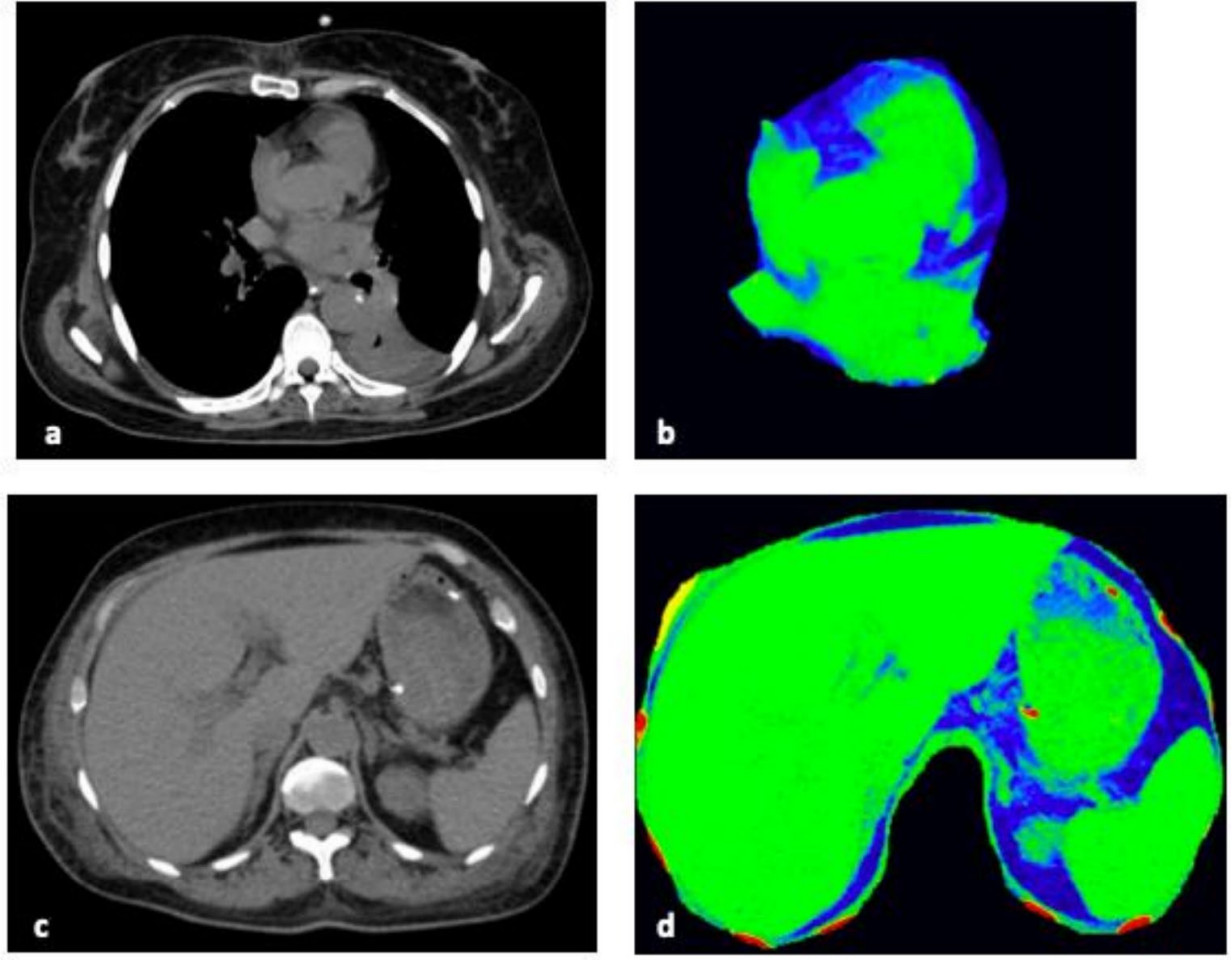
Images from Computed Tomography. Measurement site for epicardial fat at the level of the left coronary artery trunk (a). After adjustment according to Hounsfield Unit density, with fat highlighted in blue (b). Measurement site for visceral fat in the median space of the T12/L1 intervertebral disc (c). After adjustment according to Hounsfield Unit density, with fat highlighted in blue (d).

Both fat compartments were semi‐automatically segmented using predefined Hounsfield Unit (HU) thresholds: −150 to −70 HU for epicardial adipose tissue (EAT) and −190 to −50 HU for visceral adipose tissue (VAT) [22, 23] (Figure 1). The same radiologist checked intra‐observer reliability by reanalyzing 10 randomly chosen CT scans. The Intraclass Correlation Coefficient (ICC, a reliability statistic) was then calculated.

### 2.5 Statistical Analysis

We used standard statistical methods for data analysis. The Shapiro–Wilk test assessed normality. Non‐parametric data were analysed with appropriate tests and results are reported as medians and interquartile ranges (IQR).

Spearman's correlation was used to test associations among age, sex, mortality, ICU length of stay, MV days, BMI, DM, DP, CVD, HTN, EAT and VAT. Patients were assigned to deceased or surviving groups. Nonparametric tests were applied, and group comparisons used the Mann–Whitney U test for continuous variables. The Chi‐square (*χ* ^2^) test was used for categorical variables.

Logistic regression analysis was used to estimate the odds of mortality among patients with and without obesity. Univariate logistic regression was first applied to EAT, VAT, BMI, HTN, DM, CVD, age and sex as independent predictors. Variables showing statistical significance in univariate analysis were included in a multivariate model. Estimated marginal means of EAT and VAT with death as the outcome were graphically represented.

Missing data were handled using pairwise deletion, retaining all available data for each specific statistical assessment. As key exposure variables (EAT and VAT) had complete data, the impact of missingness on results was minimal. A sensitivity analysis was conducted to assess the robustness of the findings, showing no significant changes.

Analyses were conducted using Jamovi v. 2.3.26.0, Statistica v.10.0 and SPSS version 25.

## 3 Results

A total of 1237 patients with a positive COVID‐19 diagnosis were admitted to the ICU between March 2020 and December 2021. Of these, 219 underwent chest CT scans and 208 met the inclusion criteria. The sample's demographic and clinical data are presented in Table 1. When comparing the two groups (deceased vs. survivors), patients who died were significantly older (*p* < 0.001), had longer ICU stays (*p* < 0.001) and required more days of mechanical ventilation (MV) (*p* < 0.001). Conversely, survivors had a significantly higher BMI than those who died (*p* < 0.001), as patients with obesity (*p* = 0.018).

**TABLE 1:** Characteristics of the study population stratified by survived versus deceased.

	Survived (113)	Deceased (95)	*p* *
Age; years	54 [19] (113)	64 [20] (95)	< 0.001
LSH; days	22 [21] (113)	18 [14] (95)	0.278
LS ICU; days	6 [13] (113)	11 [13] (95)	< 0.001
MV	6.5 [14.3] (56)	10.5 [9.75] (58)	< 0.001
BMI; kg/m^2^	29.4 [7.9] (109)	26.9 [6.55] (92)	< 0.001
EAT; cm^2^	14.2 [11.7] (109)	16.6 [15.8] (94)	0.020
VAT; cm^2^	157 [113] (113)	134 [126] (93)	0.308
Sex; male	52.67% (69)	47.32% (62)	0.532
Obesity	64.55% (51)	35.44% (28)	0.018
DM	50.84% (30)	49.15% (29)	0.526
DP	50% (09)	50% (09)	0.700
CVD	31.25% (05)	68.75% (11)	0.054
HTN	51.02% (50)	48.98% (48)	0.404

*Note:* Data are expressed as Median [IQR] and (*n*), for continuous variables and for categorized variables are expressed as relative percentage of total and (*n*).

Abbreviations: BMI, body mass index; CV, cardiovascular disease; DM, diabetes mellitus; DP, dyslipidemia; EAT, epicardial adipose tissue; HTN, hypertension; LS ICU, length of stay in the ICU; LSH, length of stay in hospital; MV, mechanical ventilation; VAT, visceral adipose tissue.

* *p‐*value from Mann–Whitney test for continuous variables and *χ* ^2^‐test for categorized variables.

Among those who died, the EAT area was significantly larger than among survivors (*p* = 0.020), while the VAT area showed no significant difference (*p* = 0.308). There was a tendency for a smaller VAT area in deceased patients (Table 1).

When categorizing EAT and VAT in individuals with and without obesity, EAT was significantly associated with mortality among individuals without obesity (*p* < 0.01). The median EAT values were comparable between patients with and without obesity who deceased, with 17.1 cm^2^ in individuals with obesity and 16.6cm^2^ in individuals without obesity. Among survivors, however, a more pronounced difference was observed, with a median EAT of 17.5 cm^2^ in patients with obesity compared to 12.8 cm^2^ in patients without obesity (Table 2).

**TABLE 2:** Mann–Whitney test (*U*‐test) for clinical outcomes categorized into with and without obesity groups, for the variables epicardial adipose tissue (EAT) and visceral adipose tissue (VAT).

Variables	Obesity	Survived	Deceased	*p*
**EAT; cm** ^**2**^	**With**	17.5 [14.2] (51)	17.1 [13.2] (28)	0.837
	**Without**	12.8 [11.1] (59)	16.6 [15.7] (63)	< 0.001
**VAT; cm** ^**2**^	**With**	195 [118] (51)	197 [164] (28)	0.483
	**Without**	131 [121] (59)	123 [107] (63)	0.798

*Note:* Data are expressed as median [IQR] and (*n*).

Abbreviation: *p*, *p*‐value.

**FIGURE 2:**
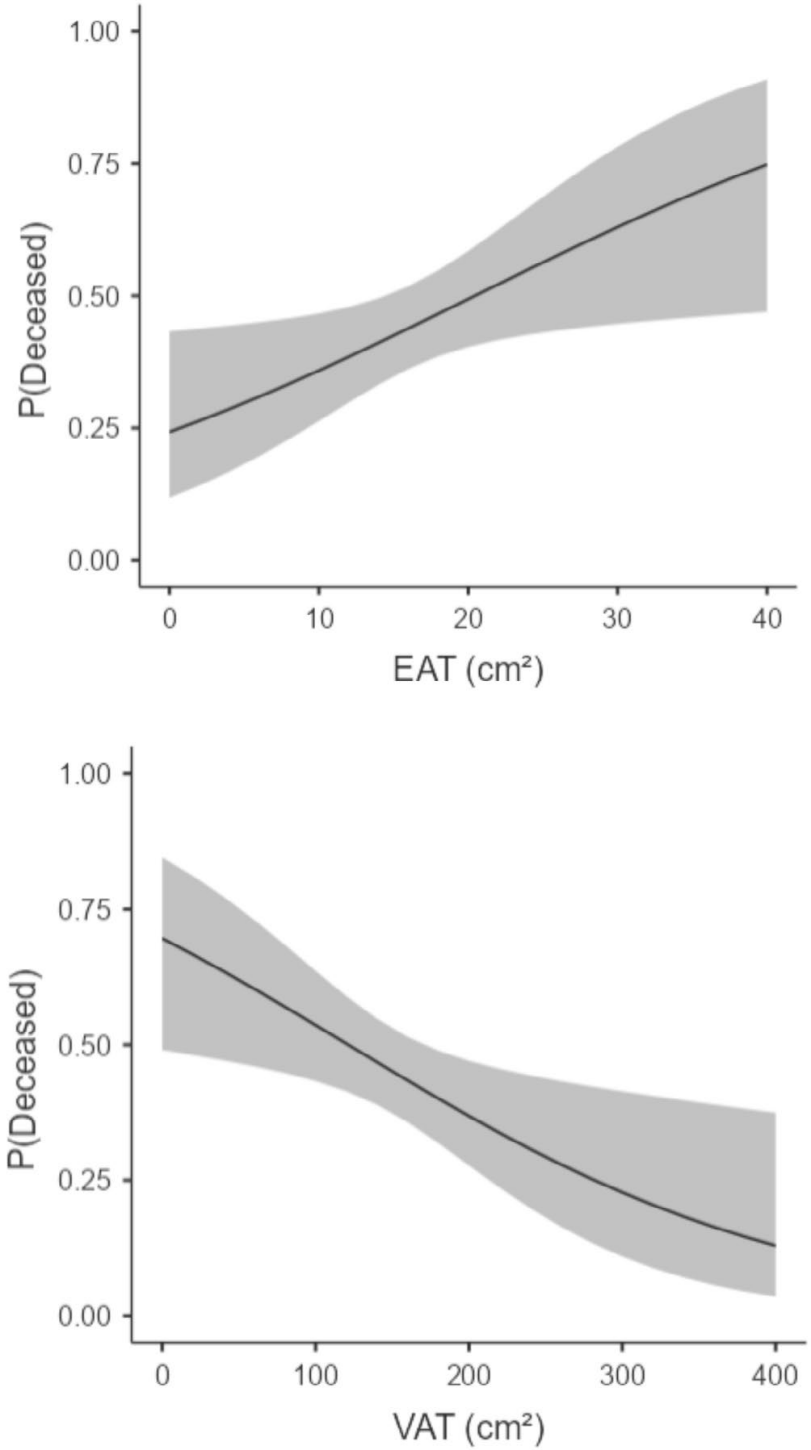
Estimated marginal means, represented by plot probability (*p*) of decease versus epicardial adipose tissue (EAT; cm^2^) and visceral adipose tissue (VAT; cm^2^).

EAT was associated with mortality (Spearman‐test = 0.144; *p* < 0.01) and was associated with an increased risk of death in individuals without obesity after adjustment for VAT, BMI and age in logistic regression analysis (Table 3). The Estimated Marginal Means analysis (Figure 2) showed that the probability of mortality increases as EAT area expands, whereas an inverse association was observed between VAT area and mortality risk—a finding that warrants careful interpretation, as discussed below.

**TABLE 3:** Logistic regression models to clinical outcomes of deceased, categorized into with and without obesity groups, to predictors epicardial adipose tissue (EAT), visceral adipose tissue (VAT), body mass index (BMI), age and as factor diabetes mellitus (DM), hypertension (HTN), cardiovascular disease (CVD) and sex.

Univariate analysis		
	OR (95% CI)	*p*
With obesity		
EAT, cm^2^	1.007 (0.970–1.050)	0.702
VAT; cm^2^	0.999 (0.994–1.000)	0.559
BMI; kg/m^2^	0.986 (0.892–1.090)	0.789
Age; years	1.061 (1.022–1.101)	0.002
Sex	1.169 (0.477–2.868)	0.733
HTN	0.454 (0.185–1.120)	0.085
CVD	10.385 (1.154–93.479)	0.037
DP	3.724 (0.324–42.834)	0.291
DM	0.997 (0.378–2.629)	0.995
Without obesity		
EAT; cm^2^	1.060 (1.015–1.107)	0.009
VAT; cm^2^	1.001 (0.997–1.010)	0.642
BMI; kg/m^2^	0.904 (0.800–1.020)	0.108
Age; years	1.032 (1.007–1.059)	0.014
Sex	0.630 (0.298–1.330)	0.226
HTN	0.989 (0.485–2.020)	0.976
CVD	1.450 (0.387–5.410)	0.582
DP	0.797 (0.270–2.350)	0.681
DM	1.495 (0.671–3.330)	0.325

Multivariate analysis				
Without obesity	Model 1		Model 2	
	OR (95% CI)	*p*	OR (95% CI)	*p*
EAT; cm^2^	1.105 (1.035–1.180)	0.003	1.097 (1.026–1.170)	0.006
VAT; cm^2^	0.995 (0.988–1.000)	0.196	0.994 (0.987–1.000)	0.113
BMI; kg/m^2^	0.894 (0.775–1.030)	0.120	0.905 (0.784–1.040)	0.169
Age; years			1.027 (0.999–1.060)	0.060

*Note:* Models: 1Adjusted for EAT, VAT and BMI conditions; 3. Adjusted for EAT, VAT, BMI and Age conditions.

Abbreviations: CI, confidence interval; OR, odds‐ratio; *p*, *p*‐value.

The ICC demonstrated excellent reliability for both EAT (ICC = 0.978, 95% CI 0.916–0.994) and VAT (ICC = 0.998, 95% CI 0.991–0.999).

## 4 Discussion

Our findings suggest that an increase in EAT area, but not VAT area, is associated with mortality in critically ill patients with COVID‐19, particularly in patients without obesity. As previous studies have shown, EAT analysis may serve as a prognostic marker for risk stratification [15]. Furthermore, our findings align with studies indicating that EAT is associated with an increased risk of death, including populations without obesity [24]. It is important to emphasize, however, that the multivariate models employed did not include disease severity scores (such as APACHE II or SOFA), inflammatory markers (such as CRP, IL‐6, or D‐dimer), or key ICU interventions (such as corticosteroid use or prone positioning). Accordingly, these results should be interpreted strictly as associations and do not establish EAT as an independent determinant of mortality.

The observed OR of 1.097 (95% CI 1.026–1.170) for EAT in the without‐obesity subgroup (Model 3, adjusted for VAT, BMI and age) represents a 9.7% increase in the odds of mortality for each additional cm^2^ of EAT area. Although this per‐unit effect size may appear modest when considered in isolation, its clinical significance becomes more apparent when interpreted within the observed distribution of EAT values in this cohort. The median EAT difference between deceased and surviving patients without obesity was approximately 3.8 cm^2^ (16.6 cm^2^ vs. 12.8 cm^2^), which corresponds to a cumulative estimated increase in mortality odds of approximately 43% (OR ≈1.097^4^). Moreover, an interquartile range increment of approximately 10 cm^2^—well within the range observed in this population—would correspond to an estimated OR of approximately 2.5, a magnitude that is clinically relevant in a critically ill cohort. These figures are broadly consistent with effect sizes reported for EAT in other cardiovascular and respiratory disease contexts. Fukushima et al. identified EAT area as the strongest CT‐derived predictor of COVID‐19 severity among various adipose tissue compartments, reporting a comparable direction of association [24]. Likewise, Erdöl et al. demonstrated that epicardial fat volume significantly predicted clinical severity of COVID‐19, with statistically significant OR values in a similar range [25]. For further contextualization, age—a widely accepted independent risk factor for mortality in critically ill COVID‐19 patients—has been associated with ORs in the range of 1.04–1.08 per year of age in large cohort studies, a per‐unit magnitude comparable to that observed for EAT per cm^2^ in the present analysis. Taken together, the OR for EAT in the without‐obesity subgroup is of clinically plausible magnitude and directionally consistent with the existing literature, supporting the biological relevance of the observed association, even while acknowledging the exploratory and hypothesis‐generating nature of this study. An important conceptual consideration concerns the interpretation of findings in individuals classified as not having obesity on the basis of BMI alone. Although BMI‐based categorization is pragmatic and widely used, it has recognized limitations with respect to body fat distribution. A BMI below 30 kg/m^2^ does not exclude excess visceral or ectopic fat accumulation, and a subset of individuals within this range may exhibit what is sometimes described as a metabolically unhealthy normal weight phenotype—characterized by disproportionate visceral adiposity, insulin resistance, and enhanced pro‐inflammatory activity, despite not meeting the conventional BMI threshold for obesity. The present finding that individuals without obesity (BMI < 30 kg/m^2^) with elevated EAT experienced higher mortality may, at least in part, reflect this phenotypic heterogeneity: excess ectopic cardiac fat may accumulate preferentially in certain individuals irrespective of overall BMI category, conferring cardiometabolic risk that is not captured by weight‐for‐height metrics alone. Integrating this conceptual framework strengthens the biological plausibility of the observed EAT–mortality association and underscores the potential value of imaging‐based fat compartment assessment—such as EAT quantification from routine CT scans—as a complementary risk stratification tool beyond BMI‐based classification.

This study found no significant association between VAT and poor outcomes in the overall sample; however, the Estimated Marginal Means analysis indicated an inverse association between VAT area and mortality risk. This finding must not be interpreted as evidence of a direct protective effect of visceral fat. In critically ill populations, a reduction in visceral adipose tissue may reflect underlying frailty, sarcopenia, or severe catabolic states—conditions in which the depletion of metabolically active tissue represents a marker of disease severity rather than a favourable metabolic profile. Critically ill patients present profound neuroendocrine and inflammatory disruptions that accelerate protein and fat catabolism, meaning adipose tissue measurements at ICU admission may reflect pre‐admission nutritional and functional status rather than any intrinsic biological property of VAT. Previous research has identified a relationship between VAT and COVID‐19 severity in non‐critically ill hospitalized patients [26, 27, 28], and an association between increased VAT and mortality in critically ill patients has also been reported, though the sample size in that study represents only 32.52% (*n* = 67) of our population [23]. The divergence between those findings and ours may be partially explained by the specific physiological context of advanced critical illness. Sarcopenia—defined by the progressive loss of skeletal muscle mass and function—has been independently associated with increased mortality in critically ill COVID‐19 patients [29] and frequently coexists with reduced visceral fat stores in severely ill individuals. Patients presenting with lower VAT at ICU admission may therefore represent a subgroup with greater pre‐existing frailty or nutritional depletion, confounding the relationship between VAT and mortality. Accordingly, the VAT‐related findings of the present study should be regarded as hypothesis‐generating and interpreted within the recognized limitations of a retrospective study conducted in a critically ill population subject to substantial metabolic derangement. Future studies incorporating simultaneous assessment of muscle mass, nutritional status, and functional capacity alongside visceral fat compartment measurements would be necessary to adequately disentangle these relationships.

The superior prognostic capability of EAT for COVID‐19 mortality may be attributed to its lack of an anatomical barrier with the myocardium, sharing the same microvascular circulation [30]. Increased EAT volume is associated with atrial fibrillation, heart failure, CVD, and worsening of respiratory infections [25, 31, 32]. Given its proximity to the myocardium, adipokines produced by EAT can reach and modulate its function, further contributing to these effects. This proximity allows EAT‐derived adipokines to modulate myocardial function, potentially increasing mortality [31]. However, some studies did not find any relationship between EAT and bad outcomes after adjusting the model to important variables [33]. The measurements used in these studies differ from the method we employed, which might contribute to the discrepant results.

The mechanisms underlying the accumulation of excess visceral fat in the heart remain unclear. However, evidence suggests that EAT behaves differently from VAT and subcutaneous fat. Some authors propose that EAT expands primarily through hyperplasia and responds distinctively to pharmacological and lifestyle interventions [34]. Additionally, studies have reported a reduction in EAT area with hypocaloric diets but not with physical activity [35].

In our study, some individuals with larger EAT areas progressed to death despite not having obesity. These findings highlight the need for further research to identify the mechanisms driving EAT expansion, its role in inflammatory diseases, and its impact on mortality and disease progression.

This study has limitations. Firstly, it is a retrospective study involving only critically ill patients, and it does not reflect the reality of all patients or pre‐ICU complications. Only patients who underwent thoracic CT were included, preventing VAT analysis in other segments, such as abdominal CT scans. Besides, there is no consensus on the best method for assessing visceral fat from CT images; therefore, variations are expected. An additional and clinically important limitation is that the multivariate models did not adjust for several potentially relevant confounders, including disease severity scores (e.g., APACHE II, SOFA), systemic inflammatory markers (e.g., C‐reactive protein, interleukin‐6, D‐dimer, ferritin), and key ICU interventions (e.g., corticosteroid administration, prone positioning, anticoagulation). The omission of these variables may introduce residual confounding and limits the interpretation of EAT as an independent predictor of mortality. The observed associations should therefore be regarded as hypothesis‐generating. A further important methodological limitation concerns the approach used to quantify abdominal VAT. In this study, VAT was estimated from a single cross‐sectional measurement at the T12/L1 intervertebral disc level on chest CT—an approach supported by precedent in the literature [21], but one that does not fully capture the total abdominal visceral adipose tissue depot. Single‐slice estimates derived from chest CT differ conceptually from VAT measurements obtained via dedicated abdominal CT or volumetric analyses spanning multiple axial levels. Prior studies reporting significant associations between VAT and COVID‐19 severity or critical illness outcomes predominantly employed abdominal CT‐derived measurements or wider scanning windows, which capture a substantially greater proportion of the total visceral fat compartment [26, 27, 28]. Accordingly, direct comparisons between the VAT‐related findings of the present study and those of prior investigations should be interpreted with caution, as methodological differences in measurement site, imaging protocol, and analytic approach may substantially contribute to discrepancies in the observed associations. This limitation should be considered when contextualizing the absence of a significant VAT–mortality association in the current cohort relative to findings from earlier studies.

Our findings suggest that EAT area is associated with mortality risk in critically ill individuals without obesity diagnosed with COVID‐19, providing prognostic information beyond BMI assessment. EAT may represent a prognostic marker for risk stratification in this population; however, given that the multivariate models did not adjust for disease severity scores, inflammatory markers, or key ICU interventions, EAT should not be characterized as an independent determinant of mortality based on the current evidence. While EAT measurement from routine CT scans may contribute to risk stratification, these associations must be interpreted with caution and validated in prospective studies with more comprehensive confounder adjustment before informing clinical practice.

Future prospective studies should explore the mechanisms underlying EAT expansion, establish standardized cutoff values that define increased risk, examine its role in inflammatory processes, and its impact on mortality and disease progression, while considering that EAT accumulation may be influenced by sedentary behaviour, lifestyle factors, and dietary habits. Understanding these relationships could help motivate lifestyle changes.

## Author Contributions

**Rebecca Baumgartner:** conceptualization, formal analysis, investigation, methodology, writing – original draft, validation, visualization. **Jéssica A. de Paula:** conceptualization, investigation, writing – review and editing. **Cleverson A. Leitão:** investigation, writing – review and editing. **Gabriela Lazzaron‐Slob:** conceptualization, investigation, writing – review and editing. **Emilton L. Junior:** supervision, writing – review and editing. **Estela I. Rabito:** methodology, project administration, writing – review and editing. All authors have read and approved the final version of the manuscript.

## Funding

The authors have nothing to report.

## Disclosure

All procedures performed in this study involving human participants were conducted in accordance with the ethical standards of the institutional and/or national research committee, and with the 1964 Helsinki Declaration and its later amendments or comparable ethical standards.

## Ethics Statement

This study was approved by the Human Research Ethics Committee of the Hospital de Clínicas, Federal University of Paraná (no. 35738620.8.0000.0096).

## Conflicts of Interest

The authors declare no conflicts of interest.

## Data Availability

The data that support the findings of this study are available on request from the corresponding author. The data are not publicly available due to privacy or ethical restrictions.
